# Disruption of the Gut Microbiome: *Clostridium difficile* Infection and the Threat of Antibiotic Resistance

**DOI:** 10.3390/genes6041347

**Published:** 2015-12-21

**Authors:** Priscilla A. Johanesen, Kate E. Mackin, Melanie L. Hutton, Milena M. Awad, Sarah Larcombe, Jacob M. Amy, Dena Lyras

**Affiliations:** Infection and Immunity Program, Monash Biomedicine Discovery Institute and Department of Microbiology, Monash University, Clayton 3800, Australia; E-Mails: priscilla.johanesen@monash.edu (P.A.J.); kate.mackin@monash.edu (K.E.M.); melanie.hutton@monash.edu (M.L.H.); milena.awad@monash.edu (M.M.A.); sarah.larcombe@monash.edu (S.L.); jacob.amy@monash.edu (J.M.A.)

**Keywords:** *Clostridium difficile*, antibiotic-associated diarrhea, microbiome, microbiota, antibiotic resistance, mobile genetic elements

## Abstract

*Clostridium difficile* is well recognized as the leading cause of antibiotic-associated diarrhea, having a significant impact in both health-care and community settings. Central to predisposition to *C. difficile* infection is disruption of the gut microbiome by antibiotics. Being a Gram-positive anaerobe, *C. difficile* is intrinsically resistant to a number of antibiotics. Mobile elements encoding antibiotic resistance determinants have also been characterized in this pathogen. While resistance to antibiotics currently used to treat *C. difficile* infection has not yet been detected, it may be only a matter of time before this occurs, as has been seen with other bacterial pathogens. This review will discuss *C. difficile* disease pathogenesis, the impact of antibiotic use on inducing disease susceptibility, and the role of antibiotic resistance and mobile elements in *C. difficile* epidemiology.

## 1. Antibiotic-Associated Diarrhea—Disruption of the Gut Microbiome

The human gut microbiome is established shortly after birth and plays an important role in human health and disease. It consists of a complex community of microorganisms that varies between individuals. Dysbiosis of the gut microbiota is associated with numerous diseases including gastrointestinal disorders such as celiac disease and inflammatory bowel disease, as well as other systemic diseases including obesity, diabetes and rheumatoid arthritis [1,2]. Of particular concern is the use of broad-spectrum antibiotics, which, in addition to treating primary infection, profoundly affect the host microbiome, disrupting the protective host microbiota. In the gastrointestinal tract, this state of dysbiosis leaves patients susceptible to infection by opportunistic bacterial pathogens, leading to a spectrum of infectious diarrheal diseases known as antibiotic-associated diarrhea (AAD) [3]. Pathogens associated with AAD include *Clostridium difficile, Clostridium perfringens. Staphylococcus aureus* and *Klebsiella oxytoca*, however, in many AAD cases, no infectious agent can be determined [3]. While antibiotic use is the primary risk factor for infectious AAD the specific antibiotics that result in disease differ depending on the causative organism. A complicating factor of AAD is that treatment typically involves additional antibiotic administration, which prevents restoration of the protective gut microbiota, thereby prolonging disease or facilitating relapse [4]. In addition to antibiotics, the use of other medications such as acid-reducing proton-pump inhibitors is associated with a predisposition to AAD [5,6,7]. This predisposition may be a result of reduced gastric acidity, allowing bacteria to survive stomach transit [8], or result in reduced immune cell function, allowing pathogen survival [9,10,11].

Overall, AAD rates in pediatric and adult populations are similar [3]. However, in the case of *C. difficile*, which is the most common causative agent of infectious AAD, age is a major risk factor for the development of infection, with disease incidence and severity escalating as age increases [12]. Other AAD risk factors relate primarily to a reduction in health status of patients prior to disease onset with many of these risk factors being more common in elderly people. *C. difficile* infection (CDI) is primarily a hospital-acquired disease identified in elderly and immunocompromised patients, however, community-associated CDI has emerged in the last decade, and infection in people previously thought to be low-risk, such as children and pregnant women, has increased [13,14,15,16]. The changing epidemiology of this disease, coupled with the emergence of strains that cause more severe infections, has prompted the Centre for Disease Control and Prevention (CDC) to define *C. difficile* as an urgent threat [17]. Unsurprisingly, research efforts internationally remain focused on *C. difficile* as the main etiologic agent of AAD.

## 2. *C. difficile* Infection and Pathogenesis

*C. difficile* is a Gram-positive, spore-forming anaerobic pathogen that is responsible for 10%–25% of AAD cases [18]. The spectrum of disease caused by *C. difficile* commonly ranges from asymptomatic carriage or mild to moderate diarrhea with abdominal cramping to profuse diarrhea and severe abdominal pain [19,20]. Less commonly, more severe conditions such as pseudomembranous colitis, toxic megacolon or colonic perforation can occur, sometimes leading to death [19,20,21]. The antibiotics most commonly associated with CDI include clindamycin, the cephalosporin family, broad-spectrum penicillins and fluoroquinolones [22].

*C. difficile* is acquired via the fecal-oral route of transmission through ingestion of the bacterial spore of the bacterium. Upon reaching the anaerobic gut niche of susceptible patients, the spores germinate into metabolically active vegetative cells that produce active toxins and induce disease in the colon [23]. The best characterized virulence determinants are the two major exotoxins, Toxin A (TcdA) and Toxin B (TcdB). These toxins inactivate Rho family GTPases, leading to disorganization of the actin cytoskeleton, cell-rounding and apoptosis of intoxicated cells [20,24]. Toxin-mediated cell death leads to the impairment of cellular tight junctions, which increases intestinal membrane permeability and facilitates inflammation [20]. Some strains of *C. difficile* produce a third toxin, CDT (*C. difficile* transferase), which may also be involved in the disease process. CDT is a binary actin-ADP-ribosylating toxin that catalyses irreversible ADP ribosylation of monomeric actin, resulting in disruption to the host cell cytoskeleton [25]. Strains capable of producing binary toxin have been associated with more severe disease in humans [26], however, the role of this toxin in disease is not clear. While the toxins produced by *C. difficile* are important for disease, other factors are also required for colonization, immune evasion and growth in the host. These factors include fibronectin binding protein, the heat-shock protein GroEL, type IV fimbriae, the surface layer (S-layer), extracellular proteases and flagella [23,26].

## 3. *C. difficile* Spores—Transmission and Recurrence of Disease

Central to host infection are *C. difficile* spores, which promote host-to-host transmission and disease recurrence within both hospitals and the community [27]. Disease relapse rates are often high with up to 30% of patients experiencing recurrence of infection with the same strain or with a different *C. difficile* strain [28,29,30,31,32,33]. Spores are the metabolically dormant and environmentally hardy form of the bacterium, exhibiting resistance to extreme temperatures, desiccation, aerobic conditions and to many disinfectants and hospital cleaning agents [34,35,36,37]. These attributes make spores difficult to eradicate from contaminated hospitals and allows them to persist within this setting for substantial time periods [38].

Spores can be transmitted from host-to-host or via the contamination of abiotic surfaces [34]. Once ingested the spore traverses the gut where cholate-containing bile salts in the intestine initiate germination [39]. The use of cecal and small intestinal extracts isolated from mice in which the host microbiome was disrupted by antibiotics was shown to enhance the germination of *C. difficile* spores, since primary bile salts are more abundant in these preparations than the secondary bile salts that are known to inhibit the growth of *C. difficile* [40]. The natural microbiota plays an important role in this stage of infection since microorganisms such as *Clostridium scindens*, which is involved in secondary bile acid synthesis, provide resistance to *C. difficile* infection in mice [41]. Although *C. difficile*-mediated disease is toxin dependent, spores are a critical component of the life cycle of this bacterium. Indeed, in mice, antibiotic treatment induces a supershedder state, with excretion of high spore numbers and concomitant heightened disease transmission observed, which may also occur in patients undergoing antibiotic treatment in a clinical setting [42].

## 4. Antibiotic Use and Resistance in *C. difficile*

Antibiotic use and resistance is linked closely to the development of CDI and the persistence of *C. difficile* as a disease-causing agent. Pseudomembranous colitis associated with the use of antibiotics, in particular clindamycin, was first reported in the 1970s [43]. Subsequent research identifying *C. difficile* as a causative agent [44,45,46] noted that strains isolated from the feces of patients were resistant to a diverse range of antibiotics including β-lactams (penicillins and cephalosporins), aminoglycosides, lincomycin, tetracyclines, and erythromycin [46]. In the 1980s cephalosporins and broad spectrum penicillins replaced clindamycin as major pre-disposing factors to *C. difficile* infection [47,48,49]. While pseudomembranous colitis continued to remain a significant healthcare issue for many years the early 2000s marked a significant turning point with the highly publicized global emergence of the so-called “hypervirulent” *C. difficile* strains, in particular type BI/NAP1/027 [50,51,52,53]. Notably, these strains had developed fluoroquinolone resistance (FQR), most likely due to overuse of this antibiotic. Genome sequence analysis of a global collection of isolates identified two genetically distinct lineages that were responsible for the epidemics, FQR1 and FQR2, both of which had identical mutations in DNA gyrase subunit A (*gyrA*) [54]. While often associated with the “hypervirulent” strains, fluoroquinolone resistance has been documented in other strain types and is not unique to ribotype 027. In these studies, mutations in the *gyrA* and/or *gyrB* genes have also been identified in non-ribotype 027 fluoroquinolone resistant isolates of *C. difficile* [55,56,57].

Investigations into *C. difficile* antibiotic resistance have revealed that multiple mechanisms for the acquisition of antibiotic resistance exist, including transposons, mobile genetic elements and various genetic mutations. Antibiotic resistance phenotypes in *C. difficile* associated with mutations in housekeeping genes have been described for antibiotics that are not commonly used in the treatment of CDI, such as fluoroquinolones. In these instances it is likely that selective pressure *in vivo* has resulted in the development of antibiotic resistance. This has been observed with antibiotics belonging to the rifamycin class of antibiotics, in particular rifampin and rifaximin, which were previously investigated as alternative CDI therapies [58], with resistance resulting from mutations in the β subunit of bacterial RNA polymerase, *rpoB* [59,60,61]. Resistance has also been observed in *C. difficile* strains isolated from patients treated with fusidic acid [62], with mutations occurring in the *fusA* gene encoding a protein elongation factor [63].

A significant proportion of the *C. difficile* genome consists of a large number of mobile elements including putative conjugative and mobilizable transposons and bacteriophages [54,64,65,66]. While plasmids are known to play an important role in the transfer of antibiotic resistance in many human pathogens [67,68] and in other clostridia [69,70], plasmids encoding antibiotic resistance in *C. difficile* have not been described. Transferable antibiotic resistance associated with tetracycline, clindamycin and erythromycin in *C. difficile* was first reported in the 1980s [71,72,73,74] and was found to occur via horizontal gene transfer of integrated chromosomal elements such as mobilizable and conjugative transposons [75,76]. The use of tetracyclines is not a common predisposing factor in CDI [12], however, resistance to this class of antibiotics is common in *C. difficile*. Tetracycline resistance in this bacterium is mediated by a ribosomal protection mechanism encoded by the *tet*(M) gene and is associated with a conjugative transposon, Tn*5397* [74,76]. Tn*5397* is closely related to the well characterized conjugative transposon Tn*916*, first identified in *Enterococcus faecalis* and subsequently found in many Gram-positive and Gram-negative bacteria, including many important bacterial pathogens [77,78]. Tn*5397* functionally differs from Tn*916* in its integration/excision module and also contains a group II intron [76]. Transposition of Tn*5397* occurs between *C. difficile* strains, *Bacillus subtilis* [79], *E. faecalis* [80] and oral *Streptococcus* species [81]. *C. difficile* may therefore act as a donor of transferrable antibiotic resistance determinants to other bacterial genera and species that occupy the same environmental or host niche. In addition to Tn*5397*, experimental and *in silico* genome analysis has shown that *C. difficile* carries a number of other Tn*916*-like transposons, some of which encode tetracycline resistance determinants and some that carry other accessory genes [64,82,83,84]. Notably, some of these Tn*916*-like derivatives encode genes predicted to encode resistance to erythromycin and β-lactams [82].

*C. difficile* resistance to the MLS_B_ family of antibiotics, including clindamycin and erythromycin, is widespread and typically conferred by the *erm*(B) gene, which encodes a 23S RNA methylase [85]. Importantly, *erm*(B) was found to be associated with epidemics caused by clindamycin-resistant *C. difficile* isolates in four hospitals in the USA in the late 1980s and early 1990s [86]. In strain 630 *erm*(B) is located on the mobilizable element, Tn*5398* [73,85]. Variations of this element are abundant in *C. difficile* with up to 17 different genetic organizations of Tn*5398*-like derivatives described [57,75,87]. Recently *erm*(B) was also found to be present on the Tn*916*-like conjugative transposon, Tn*6194* [88], which can be transferred between *C. difficile* strains and also to *E. faecalis* [89]. Although *erm*(B) primarily appears to be transferred via cell-to-cell contact through conjugative DNA transfer of transposons, a phage-mediated transduction process that may also facilitate the dissemination of this resistance gene has also been observed [90]. This can be seen in the case of Tn*6215*, a novel 13 kb *erm*(B)-carrying novel mobilizable transposon that can be transferred to recipient cells via a conjugation-like mechanism but is also able to be transduced by phage ΦC2 [90]. The complexity of *erm*(B)-carrying elements, their multiple modes of dissemination and ability to transfer within *C. difficile* strains and to bacteria belonging to other genera highlights their genetic fluidity and the potential for antibiotic resistance gene transmission to occur amongst bacteria that share the same niche.

Chloramphenicol resistance has also been identified in *C. difficile*, despite this antibiotic not being used as a treatment for CDI. This resistance phenotype is not as common in *C. difficile* as tetracycline or erythromycin resistance, but is similarly encoded on a mobile element. Chloramphenicol resistance in *C. difficile* is mediated by the *catP* gene, which encodes a chloramphenicol acetyltransferase enzyme [91]. The *catP* gene is chromosomally located in *C. difficile* and is encoded within the mobilizable transposons Tn*4453a* and Tn*4453b*. These elements are closely related to the *catP* mobilizable transposon Tn*4451*, located on the conjugative plasmid pIP401 in *C. perfringens* [92], and collectively form the Tn*4451*/*3* family of mobilizable transposons. DNA sequence analysis has provided evidence that Tn*4451*/*3*-like elements can be found in various strains of *C. difficile* and also in commensal human intestinal bacteria, including *Clostridium nexile* and *Coprococcus* species [93]. In two *C. difficile*-associated elements the chloramphenicol resistance genes have been replaced by genes putatively encoding aminoglycoside resistance or transcriptional regulators [93]. Other putative mobilizable transposons, such as Tn*6104* from strain R20291, contain genes with distant similarity to Tn*4451*/*3* but also carry other accessory genes [83]. The presence of these variants highlights the possible modular nature of these transposons, and the potential for genetic exchange between different mobile genetic elements.

Linezolid is a synthetic antibiotic from the oxazolidinone group that is used to treat serious Gram-positive infections such as pneumonia and skin and deep tissue infections. It is typically an antibiotic of last resort for infections that are resistant to several other antibiotics. Although not used in the routine treatment of *C. difficile*, a small study indicated that linezolid may protect pneumonia patients from developing CDI [94], however, *C. difficile* resistance to this antibiotic has already been observed [95]. Resistance to linezolid can be mediated by the *cfr* (chloramphenicol-florfenicol resistance) gene which encodes an RNA methyltransferase that functions by modifying the 23S rRNA, thereby conferring resistance to phenicols, lincosamides, oxazolidinones, pleuromutilins and streptogramin A antibiotics [96]. The *cfr* gene is usually carried on plasmids [96] but is chromosomally located in *C. difficile*, where it appears to be associated with a transposon similar to Tn*6218,* a novel Tn*916*-like transposon [95]. The accessory genes of other Tn*6218* variants putatively encode resistance to polyketide antibiotics, aminoglycosides (aacA-aphD) and erythromycin (erm(B)) [97]. Again, these variants provide support for the hypothesis that *C. difficile* may act as a recipient or a donor for antibiotic resistance gene transfer in the microbiota of the gastrointestinal tract.

## 5. CDI Treatment and the Threat of Antibiotic Resistance

Treatment of acute CDI usually involves oral metronidazole or vancomycin as the first-line therapy. Vancomycin and metronizadole are both effective for the treatment of mild infection, while vancomycin is superior in the treatment of severe or recurrent CDI [98,99]. Metronizadole is often the preferred therapy for mild to moderate CDI because of its low cost [99]. Fidaxomicin has also been approved for the treatment of CDI and is similar to vancomycin in treatment efficacy, although the high cost of this antibiotic precludes its routine use. However, fidaxomicin is superior to vancomycin in preventing recurrent disease and is often used in the treatment of recurrent infections or for patients with a high relapse risk [99]. Resistance *in vivo* to vancomycin or metronidazole has not been reported, however, decreased responsiveness to metronidazole for mild to moderate infection has been observed [100]. Despite a lack of resistance to these antibiotics their use in the treatment of CDI remains of concern, primarily because of the continued disruption to the protective microbiota. In addition, there is the possibility that vancomyin use, in particular, will promote resistance and the spread of resistance determinants to other microorganisms, such as *Enterococcus* species, that may subsequently have important clinical repercussions [101,102].

One of the major concerns in the treatment of CDI is disease recurrence, estimated to occur in up to 30% of patients following an initial infection but occurring in up to 60% of patients who have multiple infection episodes [12,32]. Recurrent infection is associated with continued antibiotic use and the failure of the commensal gut microbiota to be restored. For this reason, replenishment of the gut microbiota by fecal microbial transplantation has proven to be highly successful in the treatment of recurrent infection [103] and has provided new insights into the role of microbiota in CDI and in the design of new therapeutic approaches.

In 2013, the CDC published a report naming *C. difficile* as one of the top antibiotic resistant threats to public health because of the role that antibiotic treatment plays in inducing susceptibility to CDI [17]. Although *C. difficile* has not yet developed significant resistance to the antibiotics most used for CDI treatment it is highly likely that these resistance phenotypes will emerge, as has occurred through the use of clindamycin and the fluoroquinolones. The development of vancomycin resistance in *C. difficile* is deeply concerning at a global level and is more than likely to emerge; after all, vancomycin resistance was reported in *Enterococcus* species thirty years after the introduction of this antibiotic [104].

Intimately associated with the development of antibiotic resistance in *C. difficile* is the diversity of mobile genetic elements found in this bacterium, which have been identified through functional and bioinformatic studies. Many of these elements encode antibiotic resistance genes and most of these genes are located on genetic elements that are capable of lateral gene transfer. The similarity of these elements to those from unrelated bacteria, in addition to their modular nature, suggests that genetic exchange between disparate microorganisms occurs readily. This notion is not surprising since the gastrointestinal niche is an ideal environment for DNA exchange because of the close proximity of bacteria to one another. Evidence in support of this hypothesis has been obtained through *in vitro* transfer experiments, which clearly show that these elements disseminate via lateral gene transfer events, as discussed earlier. This work also confirms that *C. difficile* can facilitate the movement of various resistance genes and that it may act as both a donor and recipient in this process.

In addition to being intrinsically resistant to some classes of antibiotics, *C. difficile* encodes many specific antibiotic resistance genes or mechanisms, which allow it to proliferate in the gut when these antibiotics are being used for patient treatment. The extended use of the fluoroquinolones, for example, has promoted the worldwide dissemination of BI/NAP1/027 clones that are associated with more severe disease. The fluoroquinolone resistance phenotype of these clones during high clinical fluoroquinolone use promoted their dissemination and transmission compared to fluoroquinolone susceptible strains [50,51,52,53]. Clearly, antibiotic use poses many risks for the continued threat of CDI, primarily through the induction of susceptibility in treated patients and in the development and selection of antibiotic resistant *C. difficile* strains.

## 6. Conclusions

*C. difficile* is the major cause of AAD and a major worldwide health concern particularly in hospitals and aged-care facilities. Community-acquired *C. difficile* infection is also on the rise. A key event preceding *C. difficile* infection is the disruption of normal gastrointestinal microbiota by antibiotics that allows *C. difficile* to proliferate in the gut niche. While resistance to antibiotics currently used to treat *C. difficile* infection, such as vancomycin and metronidazole, has not been seen clinically, resistance to many other antibiotics has been observed for this pathogen. Some of the resistance genes conferring these advantageous phenotypes are chromosomally encoded and many are located within mobile genetic elements such as conjugative and mobilizable transposons. These mobile genetic elements are often related to similar entities found in other bacterial pathogens, suggesting that they may be derived from a common source and highlighting the importance of horizontal gene transfer of antibiotic resistance between different bacterial genera and species. 

The picture emerging of *C. difficile* and antibiotic resistance is clearly one of complexity. The development of antibiotic resistance in this bacterial pathogen is of concern but it is also alarming that this bacterium may act as a reservoir for the acquisition and dissemination of resistance genes to other pathogens that occupy the same host niche. Vigilant and continuing antibiotic stewardship and antimicrobial resistance monitoring are essential if we are to tackle the global health burden of this pathogen and antibiotic resistance.
